# Supplementary material for: Automated detection and quantification of two-spotted spider mite life stages using computer vision for high-throughput in vitro assays
Source: PLoS One. 2025 Dec 29;20(12):e0333253. doi: 10.1371/journal.pone.0333253 (PMC12747362; doi:10.1371/journal.pone.0333253)
Supplement: Attachment — Submitted filename: Author Response 2.docx [file pone.0333253.s002.docx]

Dear Editor or PLOS Staff:

An automated message was received on 18 November 2025 that stated the following:

*PONE-D-25-49646R1
Automated detection and quantification of two-spotted spider mite life stages using computer vision for high-throughput in vitro assays
Dr David H. Gent

Dear Dr. Gent,

We've checked your submission and before we can proceed, we need you to address the following issues:

1. Please remove any funding-related text from the manuscript.*

I do not see where any funding-related text is present in the manuscript. Can a staff member at PLOS ONE explicitly indicate where this information exists so that this can be resolved without further delay?

Thank you,

David Gent
